# Leaders, leadership and future primary care clinical research

**DOI:** 10.1186/1471-2296-9-52

**Published:** 2008-09-29

**Authors:** John Furler, Jennifer Cleland, Chris Del Mar, Barbara Hanratty, Umesh Kadam, Daniel Lasserson, Colin McCowan, Parker Magin, Caroline Mitchell, Nadeem Qureshi, Greta Rait, Nick Steel, Mieke van Driel, Alison Ward

**Affiliations:** 1 Department of General Practice, University of Melbourne, Melbourne, Australia; 2 Department of General Practice and Primary Care, University of Aberdeen, Aberdeen, UK; 3 Faculty of Health Sciences and Medicine, Bond University, Gold Coast, Australia; 4 School of Population, Community and Behavioral Sciences, University of Liverpool, Liverpool, UK; 5 Primary Care Musculoskeletal Research Centre, Keele University, Keele, UK; 6 Department of Primary Health Care, University of Oxford, Oxford, UK; 7 Division of Community Health Sciences, University of Dundee, Dundee, UK; 8 Discipline of General Practice, University of Newcastle, Newcastle, Australia; 9 Academic Unit of Primary Medical Care, University of Sheffield, Sheffield, UK; 10 Division of Primary Care, University of Nottingham, Nottingham, UK; 11 Department of Primary Care and Population Sciences, Royal Free and University College Medical School, London, UK; 12 Faculty of Health, University of East Anglia, Norwich, UK; 13 Department of General Practice and Primary Health Care, Ghent University, Belgium and Bond University, Queensland, Australia; 14 Department of Primary Health Care, University of Oxford, UK

## Abstract

**Background:**

A strong and self confident primary care workforce can deliver the highest quality care and outcomes equitably and cost effectively. To meet the increasing demands being made of it, primary care needs its own thriving research culture and knowledge base.

**Methods:**

Review of recent developments supporting primary care clinical research.

**Results:**

Primary care research has benefited from a small group of passionate leaders and significant investment in recent decades in some countries. Emerging from this has been innovation in research design and focus, although less is known of the effect on research output.

**Conclusion:**

Primary care research is now well placed to lead a broad re-vitalisation of academic medicine, answering questions of relevance to practitioners, patients, communities and Government. Key areas for future primary care research leaders to focus on include exposing undergraduates early to primary care research, integrating this early exposure with doctoral and postdoctoral research career support, further expanding cross disciplinary approaches, and developing useful measures of output for future primary care research investment.

## Background

### Primary care matters

Primary care matters to individuals, communities and Governments. For the individual, it is the entry point to health care, when and where needed, and where the vast majority of medical care happens. Primary care has taken on increasing responsibilities, for disease prevention in the 1970s and 1980s and, more recently, long-term chronic disease management. Primary care is critical in creating and sustaining the overall health status and health equity within communities [1]. A strong self-confident primary care workforce provides the best quality care and outcomes at the most cost-effective price, something of enormous interest to governments since it can help contain spiralling costs of health care [2]. To meet the increasing demands being made of it, primary care needs a strong research and knowledge base and a thriving research culture. Despite national programs aimed at building research capacity within primary care in some countries [3-6], primary care remains the intellectual and academic Cinderella of the healthcare system, often the last choice of our brightest graduates. [7] Yet there are emerging strengths in primary care research and researchers in undertaking challenging, high quality research. This nascent leadership seen within primary care research has important implications for the future of all academic clinical research. This paper argues that further strengthening primary care clinical research and its leadership is critical to ensuring that investment in health research fulfils its implicit promise of benefits to patients and communities.

### Primary care needs its own clinical research base

Primary care continues to need more research to answer essential questions: its clinical practice remains unsupported by evidence to a greater degree than in other more narrow disciplines. Starfield [8]outlined the enormous need for more research in primary care a decade ago, yet today primary care research output remains relatively low [9-11]. Evidence is thus often absent or inadequate for questions asked by practicing primary care clinicians [12].

There are special features of primary care that mean that it cannot rely solely on evidence from specialty research. In primary care problems are more diverse than those seen in specialty practice. Patients are surrounded by family, dealing with work, economic and cultural constraints. This diversity and the range of influences on disease management make much research from 'pure' controlled hospital environments less meaningful for patients and clinicians in primary care. Suffering disease at an earlier stage of evolution and where disease prevalence is lower means needing different diagnostic criteria and thresholds[13]. We need effectiveness RCTs that measure outcome in patients encountered in daily practice, rather than the purer efficacy trials in which co-morbidity is excluded [14]. Primary care clinicians in many countries have responsibility for a defined practice population. Researching the trade-offs involved when balancing the needs of individual patients and community demands pragmatic adjustments of study design. Examples of such study designs are found in Table 1.

**Table 1:** Extraordinary potential of primary care research

**Innovative and pragmatic study designs and topics**	**Examples**
Ground breaking observational research	Discovering the infective nature incubation period of Hepatitis A [31]
"Stable" prospective cohort studies	Screening for Hypertension [32]
"Stable" retrospective cohort studies	Natural history of Hypertension[33]
Large cross-sectional databases	Developing a cardiovascular risk assessment tool that is more relevant for the UK population [34]
"Pragmatic" Cluster randomised controlled trials	Secondary prevention of CHD [35]
"Pragmatic" systematic reviews	Effect of guidelines on clinical practice [36] ; Usage of computers in consultations [37]; Role of antibiotics in sore throat [38]
"Pragmatic" factorial design	Laboratory test reminders [39]
Primary qualitative research	Chronic care models in primary care [40]; Experience of acute illness [41]
Secondary qualitative research: meta-synthesis	Perception of family history in chronic disease [42]; diabetes and diabetes care [43]
Robust economic evaluation alongside trials	Hospital at home [44]; Endoscopy for dyspepsia [45]
Process evaluation of randomised trials and complex interventions	Self management of inflammatory bowel disease [46]; Integrated heart care [47]
Complexity theory in understanding chronic illness	Lived experience of diabetes in the community [48]
Guideline evaluation & implementation; translational research	Trial of asthma guidelines [49]; Critical features of the implementation of clinical guidelines [50]
Tackling inequalities	Tuberculosis screening [51]
Evaluating primary prevention	Exercise program in primary care [52]
Early intervention in the natural history of medical conditions	Bell's palsy [53]; Conjunctivitis [54]

### Emerging strengths and leadership in primary care clinical research

Primary care researchers are developing skills and strength in tackling research topics of relevance to clinicians, patients and communities, through leading cross-disciplinary research programs and collaborations with other medical disciplines, basic and social sciences, health services researchers and policy makers[15]. Our research can lead to direct changes in practice, unlike much basic bench-top or tightly controlled experimental research. Testing effectiveness (rather than efficacy) means we have a better idea of the outcome in real life of interventions within the complexity of health care [16], and a much better chance of incorporating issues of reach, access and equity. The place of collaborative networks of clinician researchers linked to academic centres has been critical in this[17]. Examples of ground breaking primary care research in these areas are also included in Table 1. The emergence of this research enterprise in response to these unique features of primary care provides an opportunity for primary care researchers to lead the future development of clinical research. Leadership must come from the most talented researchers within primary care, trained in advanced research skills and engaged with communities, clinicians, international networks of fellow researchers, government and funders.

### Primary care research could lead the revitalisation of academic medicine

Academic clinical medicine the "capacity of the health care system to think, study, research, discover, evaluate, teach, learn and improve" [18]), has recently been described as in jeopardy [19], at a crossroads [20]and in need of revitalisation [18]. This imminent crisis in clinical research lies in its failure to keep pace with basic sciences, creating a bottleneck in the translation of evidence into health benefit for patients, and in failing to grapple with research questions of most importance to clinicians and patients. What is needed is better understanding of those problems ("basic observational clinical research"[21] incorporating inter and trans-disciplinary perspectives), trials of interventions designed from this, and translational research to put such wisdom into practice. Revitalising academic medicine along these lines will require leadership [22]. Primary care research leaders are well placed to provide this.

### Leadership and renewing investment in primary care research

Clinical research in primary care is important but is complex and demanding and requires continuing support. Leadership from within the community of researchers is important to ensure that focus is not lost, that a strong link with our clinical and community base is maintained, and that investment creates a sustainable base for the future.

Focus is easily lost. While national research capacity building programs in primary care have provided protected time for a small number of early researchers with opportunities to build skills and research experience, there are tensions inherent in this. Governments may be attracted to funding workforce and health services research at the expense of clinical questions. Similarly, engagement with clinicians and patients needs nurturing. Studies which engage practitioners during the development and conduct of research are more likely to reach a satisfactory and clinically relevant conclusion [23]. Recent investment in primary care research networks has helped generate research directly relevant to primary care [24] trying to overcome the sense that primary care is simply research "substrate". However other tensions have emerged. Support for research networks is in transition. In England, for example, the relatively new national Primary Care Research Network's main goal is to increase the number of patients involved in clinical studies [25]. This is a shift in focus from research capacity building amongst primary care practitioners to improving accrual rates in studies. Many networks have had to be resourceful in maintaining some degree of capacity development as well as a focus on clinical research. Leadership will be important in the future to navigate these tensions. In other countries, such as Australia, practice-based research networks are in their infancy and have not yet attracted dedicated funding from government. This makes engaging clinicians and retaining the vital link between research and practice much more difficult. It will require primary care leadership to secure the support that will ensure their viability.

Building a sustainable base for primary care research will also require leadership. Primary care research started late. It still lags badly in the highly competitive race, and, accordingly is seen as less respectable. Bright young minds that crave certainty are less attracted to it than more narrowly focused disciplines. Intercalated research years for medical students succeed in attracting students into research, but rarely into primary care, although this is feasible[26]. Here is an area for focus. Effort here must be linked with securing sustainable, not short term, funds for more and more flexible postgraduate scholarship and fellowship opportunities. These can be the building blocks to create meaningful and productive career paths for research-engaged clinicians as well as further strengthen the cross-disciplinary nature of emerging academic departments of primary care.

Future leaders will need to marshal evidence and passion in arguing for the potential of primary care research to meet the pressing needs of our communities. To be accountable for future investment we need data about research output of academic departments of primary care and general practice. Primary care journals are now achieving indexing status making publications more searchable but this raises questions about appropriate benchmarks for academic staff. An international clearing house of primary care evidence, publications and research reports has been suggested [27]. Case studies illustrating how Departments innovate and build capacity locally from a mix of service, teaching and research staff will help highlight gaps in support for future investment. Measuring output also highlights the tension between journal impact factors and achieving societal impact. Primary care can lead in developing new measures of appropriate impact for research [28], with implications for academic medicine more broadly. All these are important issues where advocacy from primary care research leaders can spark awareness of the intellectual challenges of primary care and how primary care research can make a difference.

Leadership in the past has been provided by a few who arose *ad hoc*, but future international primary care leadership needs active planning, development and promotion. The Brisbane Initiative (BI) is an example of this. It began in 2002, bringing together primary care academics committed to fostering leadership skills in early and developing career researchers, from the UK, Europe, the USA and Australia. The aim is to attract and support the most talented researchers in a senior research career[29]. Strategies within the initiative include cooperative development of research educational resources, the development of Expert Groups and Think Tanks, fellowships and visiting traineeships, and support of small, international peer learning set cohorts aimed at postdoctoral primary care researchers [30]. The BI model provides a structure to identify and support future leaders, building on the successes of research leaders going before them, while fostering collaborative learning across international boundaries. It also builds an important link between local, community-based research and the global discipline of primary care research.

## Conclusion

The last decade has seen important investment in primary care research capacity. Primary care research has made great advances and its strengths are increasingly apparent. This is heartening for clinicians and communities. They see research effort that is starting to address important questions they have about clinical care. It is also of particular interest to Governments and funders in the face of changing patterns of disease and health care and unsustainable health care costs. However, skilled leadership will be important in securing a new wave of investment in primary care research that ensures this momentum is not lost (see Table 2). We must nurture our strengths, retaining strong links to practice, clinicians and patient organisations and championing inter-disciplinary collaborative approaches in our work. We must expose students early to research in primary care. We must also develop ways of showcasing our achievements, assembling robust evidence of the important academic challenges in primary care, of the implications of these challenges for health and health care more widely, and of our growing capacity to address them. Our brightest undergraduates, politicians, public servants and media editors must see that primary care research is where important and exciting work is being done.

**Table 2:** Summary points

• Primary care needs a strong knowledge base to provide the best quality care and respond to the increasing demands being made of it.
• Investment in primary care research capacity has produced innovative research approaches and relevant practical research that addresses the concerns of practitioners and patients, in a cost-effective manner, although less is known of the effect on overall research output.
• The growing strength of primary care research means it can spearhead a revitalisation of academic medicine. Research leadership is needed to maintain the focus and build on the strengths of academic primary care to help create a sustainable research base and a thriving research culture.
• Critical areas of focus are engaging undergraduate students into primary care research, retaining strong links with practice and communities, strengthening interdisciplinary and collaborative research, building flexible career paths for primary care researchers and creating structures for measuring and reporting output.

## Competing interests

CDM and AW are involved in managing and developing the Brisbane Initiative. All the other authors are participants in the 2007 cohort of the Brisbane Initiative. All the authors are engaged in primary care research.

## Authors' contributions

The topic of the paper was developed through face to face discussion between a cohort of Brisbane Initiative participants in Oxford in September 2007 and subsequent email correspondence. JF wrote the first draft and is guarantor. All of the authors contributed to the writing of the paper and approved the final draft.

## Pre-publication history

The pre-publication history for this paper can be accessed here:


